# Emergence of Pathogenicity in Lagoviruses: Evolution from Pre-existing Nonpathogenic Strains or through a Species Jump?

**DOI:** 10.1371/journal.ppat.1005087

**Published:** 2015-11-05

**Authors:** Pedro José Esteves, Joana Abrantes, Stéphane Bertagnoli, Patrizia Cavadini, Dolores Gavier-Widén, Jean-Sébastien Guitton, Antonio Lavazza, Evelyne Lemaitre, Jérôme Letty, Ana Margarida Lopes, Aleksija S. Neimanis, Nathalie Ruvoën-Clouet, Jacques Le Pendu, Stéphane Marchandeau, Ghislaine Le Gall-Reculé

**Affiliations:** 1 InBIO—Research Network in Biodiversity and Evolutionary Biology, CIBIO, Campus de Vairão, Universidade do Porto, Vairão, Portugal; 2 Departamento de Biologia, Faculdade de Ciências da Universidade do Porto, Porto, Portugal; 3 CESPU, Instituto de Investigação e Formação Avançada em Ciências e Tecnologias da Saúde, Gandra, Portugal; 4 UMR 1225, INRA, Toulouse, France; 5 INP-ENVT, University of Toulouse, Toulouse, France; 6 Proteomic Unit, Istituto Zooprofilattico Sperimentale della Lombardia e dell’Emilia Romagna “Bruno Ubertini”, Brescia, Italy; 7 Department of Pathology and Wildlife Diseases, National Veterinary Institute, Uppsala, Sweden; 8 Department of Studies and Research, National Hunting and Wildlife Agency (ONCFS), Nantes, France; 9 Virology Unit, Istituto Zooprofilattico Sperimentale della Lombardia e dell’Emilia Romagna “Bruno Ubertini”, Brescia, Italy; 10 Avian and Rabbit Virology Immunology Parasitology Unit, Ploufragan-Plouzané Laboratory, French Agency for Food, Environmental and Occupational Health & Safety (Anses), Ploufragan, France; 11 European University of Brittany, Rennes, France; 12 Inserm U892; CNRS, UMR 6299University of Nantes, Nantes France; The Fox Chase Cancer Center, UNITED STATES

## Introduction

Emergence of pathogenic viruses is of great concern, although the underlying mechanisms for emergence remain often poorly understood. RNA viruses are frequently implicated and recent examples include viruses within *Orthomyxoviridae*, *Flaviviridae*, or *Coronaviridae* [1–3]. Within *Caliciviridae*, the *Lagovirus* genus is particularly intriguing because it has generated viruses of exceptional pathogenicity on several occasions within the past 40 years. The genus *Lagovirus* encompasses two pathogenic viruses, Rabbit hemorrhagic disease virus (RHDV) affecting European rabbit (*Oryctolagus cuniculus*), and European brown hare syndrome virus (EBHSV) affecting Brown, Mountain, and Italian hare (*Lepus europaeus*, *L*. *timidus*, and *L*. *corsicanus*) [4]. These two viruses show a similar structure and ~70% homology [5–15]. They cause two distinct diseases, RHD (rabbit hemorrhagic disease) and EBHS (European brown hare syndrome), that emerged in the late 1970s to early 1980s [16–17]. Both cause high mortalities and impose a heavy economic burden on the rabbit industry and game animal management. They have also contributed to declines of wild leporid populations throughout Europe, resulting in major ecological impact in natural ecosystems where leporids are key species [18–25].

RHD was first detected in China in 1984, apparently in rabbits imported from Germany [16], suggesting that RHDV originated in Europe. Rabbit lagoviruses consist of pathogenic viruses (RHDV) and related, but genetically divergent, nonpathogenic viruses [26–34]. Phylogenetic analyses of pathogenic RHDV strains show three distinct groups: the classic RHDV with the genogroups G1–G5 [27,35–55], the antigenic variant RHDVa/G6 [35,56–61], and RHDV2/RHDVb [62–77]. The RHDV and RHDVa are phylogenetically related and differ from RHDV2 by more than 15% in nucleotide diversity. The RHDV strains have emerged at different times: RHDV was first isolated in 1984 [16] and RHDV2 in 2010 [62]. RHDV2 was identified in France and has since spread throughout other Western European countries, replacing the circulating strains in France and the Iberian Peninsula [64,67,71,76], while in Italy, it currently cocirculates with the “original” strains [64].

EBHS was first reported in Sweden in 1980 [17] and later found in other European countries [11,78–85]. It may have emerged earlier, as suggested by descriptions of hares with lesions consistent with the disease in 1976 in England [86]. Otherwise, antibodies against the virus have also been found in archived sera [87], and the virus was detected by RT-PCR in samples collected in Sweden before 1980 [88].

Two competing hypotheses can be put forward to explain RHDV and EBHSV origin and the emergence of RHDV2: 1) the evolution from pre-existing nonpathogenic viruses circulating in European leporids; 2) a species jump. The first hypothesis is shared by several authors and originates from the detection of anti-RHDV antibodies in rabbit blood samples collected before the first documented outbreak in Europe and Australia [89–92] and later in the characterization of different nonpathogenic viruses in European rabbits [26–34]. However, this hypothesis has not been confirmed and fails to explain the abrupt emergence of high pathogenicity on several occasions in a short period of time. Notably, the pathogenic and nonpathogenic viruses are phylogenetically separated and display ~20% of nucleotide divergence in the capsid gene [26–34], suggesting that the pathogenic forms did not directly originate from the nonpathogenic ones. Nevertheless, nonpathogenic strains have not been exhaustively characterized in European leporids, which may explain why the ancestors of pathogenic strains have not yet been found. The second hypothesis involves a species jump from species sympatric with European leporids, either native or previously introduced. Among these species, Eastern cottontail (*Sylvilagus floridanus*), a leporid native to North America, would constitute a worthwhile species. Although no data is available on the presence of original lagoviruses, they likely would have caused asymptomatic infection in its natural host, with a course of infections similar to what occurs with myxoma virus, benign in *Sylvilagus* species, but lethal in the European rabbit following a species jump [93]. Indeed, it is intriguing that both RHDV and EBHSV emerged at around the same time, coinciding with the introduction of the Eastern cottontail in Europe. Large numbers of introductions of Eastern cottontails by hunters occurred in Europe from the 1960s, but because they were illegal, these introductions are poorly documented. The first known introduction attempt from the United States was in 1966 in Italy. This was followed by massive introductions involving thousands of animals, especially in Italy and France. It is highly likely that localized introductions still occur, as suggested by the existence of cottontail breeders in France.

In the Po valley in Italy, where Eastern cottontails are invasive and widespread, a serological study showed that 18% and 33% of them carry antibodies detected by both anti-EBHSV and anti-RHDV serological tests, proving the susceptibility of the species to lagoviruses [94]. Moreover, recent documentation of RHDV strains in Iberian hares (*L*. *granatensis*) with lesions compatible with RHD [95], demonstration of the capacity of RHDV2 to infect Sardinian Cape and Italian hares (*L*. *capensis mediterraneus* and *L*. *corsicanus*, respectively) causing RHDV-like disease [65,70], and the experimental infection of cottontails by EBHSV [94], show the feasibility of species jumps of lagoviruses between leporid species.

**Fig 1:**
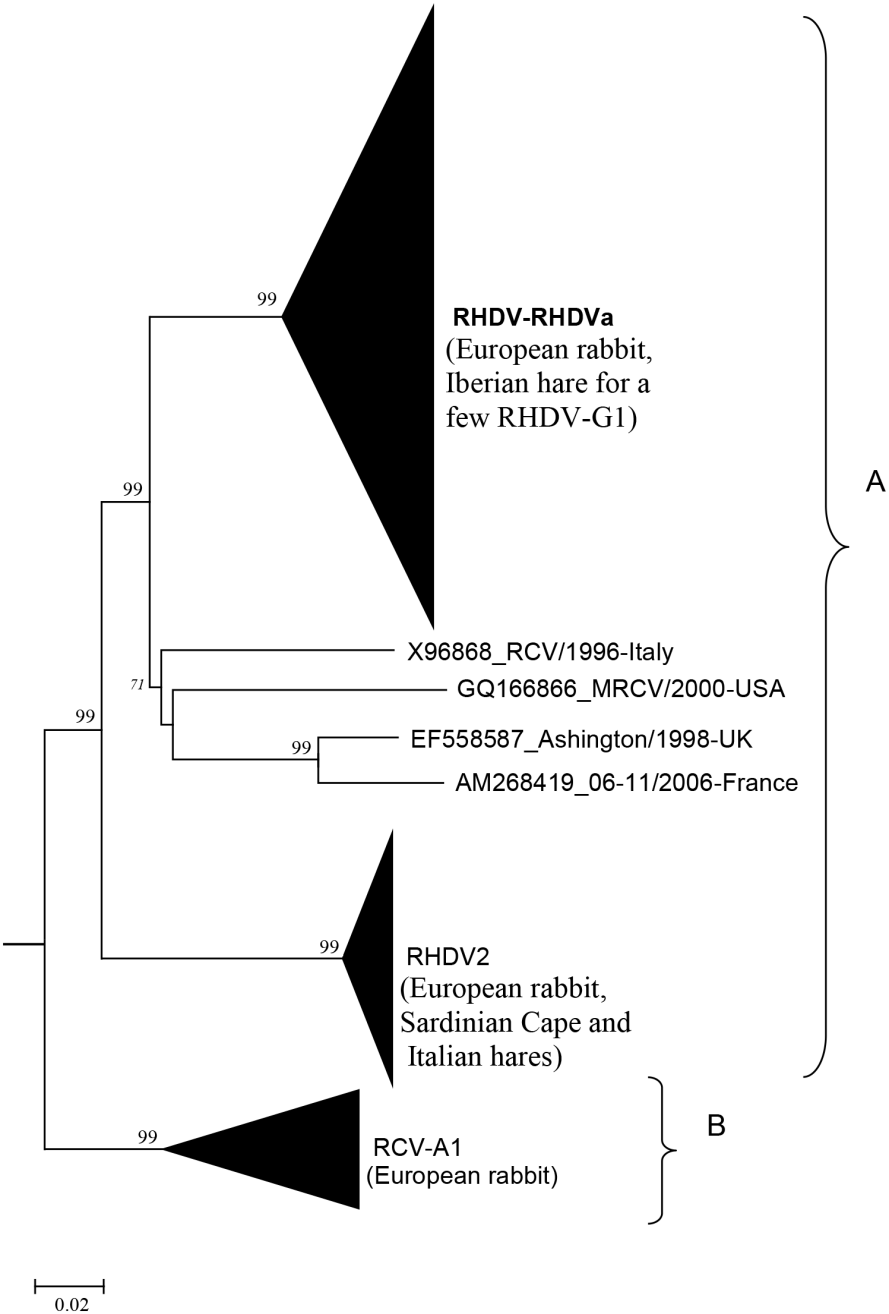
Possible origin of European rabbit (*O*. *cuniculus*) lagoviruses according to the hypothesis of a species jump. A) Lagoviruses that may share common ancestors following several species jump(s), B) Nonpathogenic viruses that have evolved in European rabbit for a long time. Phylogenetic tree (Neighbor-joining method) derived from 303 rabbit lagovirus sequences of the VP60 gene available on public databases (May 2015). The pathogenic RHDV, RHDVa, RHDV2, and the nonpathogenic RCV-A1 branches are collapsed; the name of the leporid species where these strains were isolated is given in brackets. X96868_RCV/1996-Italy, GQ166866_MRCV/2000-USA, EF558587_Ashington/1998-UK, and AM268419_06-11/2006-France are nonpathogenic strains isolated in the European rabbit. Percentage greater than 70% of replicate trees in which the associated taxa clustered together in the bootstrap test (500 replicates) are given at major branch nodes. The EBHSV strain GD (Z69620) was used as an outgroup to root the tree. Similar clustering was observed in several recent works [63,64,66,70,74].

We therefore suggest that European leporids carry lagoviruses of two distinct origins (Fig 1): nonpathogenic strains that have evolved with these species for a long time and a second group including pathogenic strains that possibly emerged following species jumps from *S*. *floridanus* and that have then evolved in European leporids. Pathogenic strains may be pure cottontail viruses or recombinants of cottontail viruses and nonpathogenic viruses of European leporid species. Indeed, recombination in RHDV is reported [96,97], and the recent documentation of recombination events between genome regions encoding the capsid and VP10 structural proteins of RHDV2 and the nonstructural proteins from nonpathogenic or pathogenic G1 lagoviruses suggests that recombination could have had an important role in the lagovirus evolution [74].

Evaluation of potential emergence of new pathogenic lagoviruses from the nonpathogenic strains circulating among native European leporid species and *Sylvilagus* species, as well as characterization of the genetic determinisms of pathogenicity, are key to identify the mechanisms of disease emergence and may help to evaluate the possibility of emergence of other highly pathogenic lagoviruses.
